# Supplementary material for: Phosphatidylinositol Monophosphates Regulate the Membrane Localization of HSPA1A, a Stress-Inducible 70-kDa Heat Shock Protein
Source: Biomolecules. 2022 Jun 20;12(6):856. doi: 10.3390/biom12060856 (PMC9221345; doi:10.3390/biom12060856)
Supplement: Supplementary file 1 [file biomolecules-12-00856-s001.zip › biomolecules-1707333-supplementary.pdf]

Supplementary Materials

Phosphatidylinositol monophosphates regulate the membrane localization of HSPA1A, a stress-inducible 70-kDa heat shock protein

Larissa Smulders [1#¶], Rachel Altman [1#], Carolina Briseno [1], Alireza Saatchi [1], Leslie Wallace [1], Maha AlSebaye [1], Robert V. Stahelin [2] and Nikolas Nikolaidis [1,*]

[1] Department of Biological Science, Center for Applied Biotechnology Studies, and Center for Computational and Applied Mathematics, College of Natural Sciences and Mathematics, California State University Fullerton, Fullerton, CA 92834-6850; Larissa.Smulders@age.mpg.de (L.S.); raltman18@csu.fullerton.edu (R.A.); cbriseno89@csu.fullerton.edu (B.C.); Ali.Saatchi17@csu.fullerton.edu (A.S); Lannewallace@csu.fullerton.edu (L.W.); meyhab@csu.fullerton.edu (M.A.)
[2] Department of Medicinal Chemistry and Molecular Pharmacology and the Purdue University Cancer Center, Purdue University, West Lafayette, IN, 47907, USA; rstaheli@purdue.edu (R.V.S.)
[#] Correspondence: These authors contributed equally
[¶] Correspondence: Current address: Max Planck Institute for Biology of Ageing, Joseph-Stelzmann-Straße 9B, 50931 Cologne, Germany
[*] Correspondence: Correspondence: e-mail: nnikolaidis@fullerton.edu; Tel.: 001-657-278-4526

Supplementary Figures

Figure S1 Confocal microscopy image analyses to quantify HSPA1A's PM localization. Representative images of HeLa cells expressing GFP-HSPA1A (first column from the left; green pseudocolor) stained with WGA-FA555 PM stain (second column; red pseudocolor) and DAPI nucleus stain (third column; blue pseudocolor). Overlay images showing all stains as different pseudocolors are provided in the fourth (using green pseudocolor for GFP) and fifth column (using cyan pseudocolor for GFP). The localization of HSPA1A was documented at either control conditions (37°C; top row) or heat-shocked conditions (1h at 42°C followed by 8h at 37°C; bottom row). Scale bar=10μm. (B) Graphical representation of the procedure used to quantify the PM localized HSPA1A in a selected cell (inlet). The procedure starts by opening all the images that belong to a set with ImageJ followed by (i) window synchronization. The next step will measure background intensity. Start with the image that shows HSPA1A, select bean shape drawing tool, and (ii) draw a shape of approximately cell size that doesn't include any cells. (iii) Use "m" on keyboard to calculate intensity of selected area. Save numbers; these would represent the background values. The next step will measure cytoplasmic intensity. Select the bean shape drawing tool and (iv) draw around the nucleus and around the cell. (v) Fit region of interest (ROI) to perfectly fit the outline of the cell by changing the thresholds and (vi) press "m" to measure the intensity of the cytoplasm. Save the numbers. The next step will measure the plasma membrane intensity. Select the bean shape drawing tool and (vii) draw a line right next to the outline of the cell using the PM stain for reference (hold down "alt" on the keyboard and start drawing a line 6 pixels within the previous cell outline). The space between the two lines is the PM (should correspond to the WGA line). (viii) Press "m" to measure the intensity of the PM. Save numbers.

Figure S2. Cell surface biotinylation reveals that HSPA1A's PM localization depends on the availability of PI(4)P and PI(3)P. The figure presents the full Western blots shown (A) in Figure 3A and (B) in Figure 3B. (A) The first panel from the top shows total protein. The nitrocellulose membranes were stained with the Reversible Protein Stain Kit (Pierce). The same blots were then stained with multiple antibodies. The second panel shows the result of the OmicsLink™ Anti-GFP Tag Antibody Mouse Monoclonal IgG1 [(CGAB-GFP-0050); 1:1000]. This antibody detects a protein band of approximately 30kDa (empty GFP), one band of approximately 98kDa

(GFP-HSPA1A), and a band of approximately 55kDa (GFP-P4M-SidMx2). The third panel shows staining with the anti-HSP70 Monoclonal Antibody [(mouse IgG (Dilution 1:1000) clone #C92F3A-5 (detects a protein around 74kDa (native HSPA1A)]. The fourth panel shows the result of THE™ beta actin antibody, GenScript [(mouse mAb; A00702) 1:1000, which detects endogenous actin (band 45kDa). The fifth panel shows the Na+/K+ ATPase α (ATP1A1) antibody RabMAb® [(EP1845Y); (2047-1); 1:1000]. This antibody detects an endogenous protein around 112kDa. In all blots M corresponds to molecular size marker (Fisher BioReagents™ EZ-Run™ Prestained Rec Protein Ladder; approximate sizes shown on the right side of the blots). (B) The first panel from the top shows total protein. The second panel shows the result of the Anti-c-Myc Antibody (9E10) [monoclonal IgG (Dilution 1:1000)- detects a protein of approximately 74kDa (HSPA1A-myc)]. The third panel shows staining with the anti-GFP antibody, which detects a protein band of approximately 30kDa (empty GFP) and a band of approximately 98kDa (GFP-EEA1). The fourth and fifth panes show the results of the anti-actin and anti-ATPA1, which detect endogenous actin (band 45kDa) and ATP1A1 (band 112kDa). In all blots M corresponds to molecular size marker (Fisher BioReagents™ EZ-Run™ Prestained Rec Protein Ladder; approximate sizes shown on the right side of the blots).

Figure S3. Isolation of total PM proteins of cells expressing (A) GFP-HSPA1A alone and (B) GFP-HSPA1A together with GFP-P4M-SidMx2. Equal volumes of crude fractions corresponding to the nucleus (N), cytosol (C), total organelle membranes (O), and plasma membrane (P) are shown. The nitrocellulose membranes were stained with the Reversible Protein Stain Kit (Pierce) followed by western analysis using the following antibodies: the OmicsLink™ Anti-GFP Tag Antibody Mouse Monoclonal IgG1 [(CGAB-GFP-0050) and the Na+/K+ ATPase α (ATP1A1) antibody RabMAb® [(EP1845Y); (2047-1)]. The anti-GFP antibody detects overexpressed GFP-HSPA1A (98kDa) and GFP-P4M-SidMx2 (54kDa). The second antibody (ATP1A1) detects an endogenous protein around 112kDa. M: molecular size marker (BioRad Dual Color Protein Ladder; approximate sizes shown on the right side of the blots).

Figure S4. Cell surface biotinylation reveals that HSPA1A's PM localization depends on the availability of PS, PI(4)P, and PI(3)P, but not PI(4,5)P_2. The first panels from the top show total protein stain [Reversible Protein Stain Kit (Pierce)]. The second panels show the result of the Anti-c-Myc Antibody (9E10) [monoclonal IgG (Dilution 1:1000)- detects a protein of approximately 74kDa (HSPA1A-myc)]. The third panels show the result of the OmicsLink™ Anti-GFP Tag Antibody Mouse Monoclonal IgG1 [(CGAB-GFP-0050); 1:1000]. This antibody detects a protein band of approximately 30kDa (empty GFP), a band of approximately 54kDa (GFP-P4M-SidMx2, LactC2-GFP, GFP-PLCδ), and a band of approximately 98kDa (GFP-EEA1). The fourth panels show the result of THE™ beta actin antibody, GenScript [(mouse mAb; A00702) 1:1000, which detects endogenous actin (band 45kDa). The fifth panels show the Na+/K+ ATPase α (ATP1A1) antibody RabMAb® [(EP1845Y); (2047-1); 1:1000]. This antibody detects an endogenous protein around 112kDa. M: molecular size marker (Fisher BioReagents™ EZ-Run™ Prestained Rec Protein Ladder; approximate sizes shown on the left side of the blots).

Figure S5. Cell surface biotinylation reveals that HSPA1A's PM is decreased after heat-shock in cells treated with Phenyl arsine oxide (PAO) and wortmannin (Wort). The nitrocellulose membranes were stained with the Reversible Protein Stain Kit (Pierce; first panel from top) followed by western analysis using the following antibodies: anti-c-Myc Antibody (9E10) [monoclonal IgG (Dilution 1:1000)- detects a protein of approximately 74kDa (HSPA1A-myc tagged]; THE™ beta actin antibody, GenScript [(mouse mAb; A00702) 1:1000; and the Na+/K+ ATPase α (ATP1A1) antibody RabMAb® [(EP1845Y); (2047-1); 1:1000. These antibodies detect endogenous actin (band 45kDa) and ATP1A1 (band 112kDa). M: molecular size marker (Fisher BioReagents™ EZ-Run™ Prestained Rec Protein Ladder; approximate sizes shown on

the right side of the blots). The bottom panel shows the quantification of the antibody detected signals of the HSPA1A-myc in the absence (DMSO) or presence of PAO or wortmannin. The values are presented as a ratio between the biotinylated (PM) fraction and the total cell lysate. Densitometry values are averages of three independent experiments (n=3). These values were normalized to controls (control set to 100%) and the standard deviation was scaled accordingly. Center lines show the medians; whiskers extend 1.5 times the interquartile range from the 25th and 75th percentiles; crosses represent sample means.

Figure S6. Effect of treatment with GSK-A1 on the PM localization of HSPA1A and several lipid biosensors. Representative images of HeLa cells expressing GFP-HSPA1A (first two rows), GFP-P4M-SidMx2 (second two rows), GFP-PLCδ-PH (third two rows), Lact-C2-GFP (fourth two rows), and GFP-EEA1 (fifth two rows). In all cases, the PM was stained with WGA-FA555 and the nucleus with DAPI. The PM localization of the proteins was documented at either control conditions (37°C; left four columns) or heat-shocked conditions (1h at 42°C/ 8h at 37°C; right four columns). Negative control cells were treated with DMSO. Scale bar=10μM.

Figure S7. The subcellular distribution of EEA1, the biosensor for PI(3)P, is unaffected by the Sac1 and INPP5E enzymes. Representative images of HeLa cells expressing GFP-EEA1 with RFP-tagged PJ vectors and DAPI nucleus stain. The localization of EEA1 was documented at either control conditions (37°C; left two columns) or heat-shocked conditions (1h at 42°C/ 8h at 37°C; right two columns). Negative control cells contain either the vehicle alone (Lyn11) or the inactive vector (Dead). Scale bar=10μM.

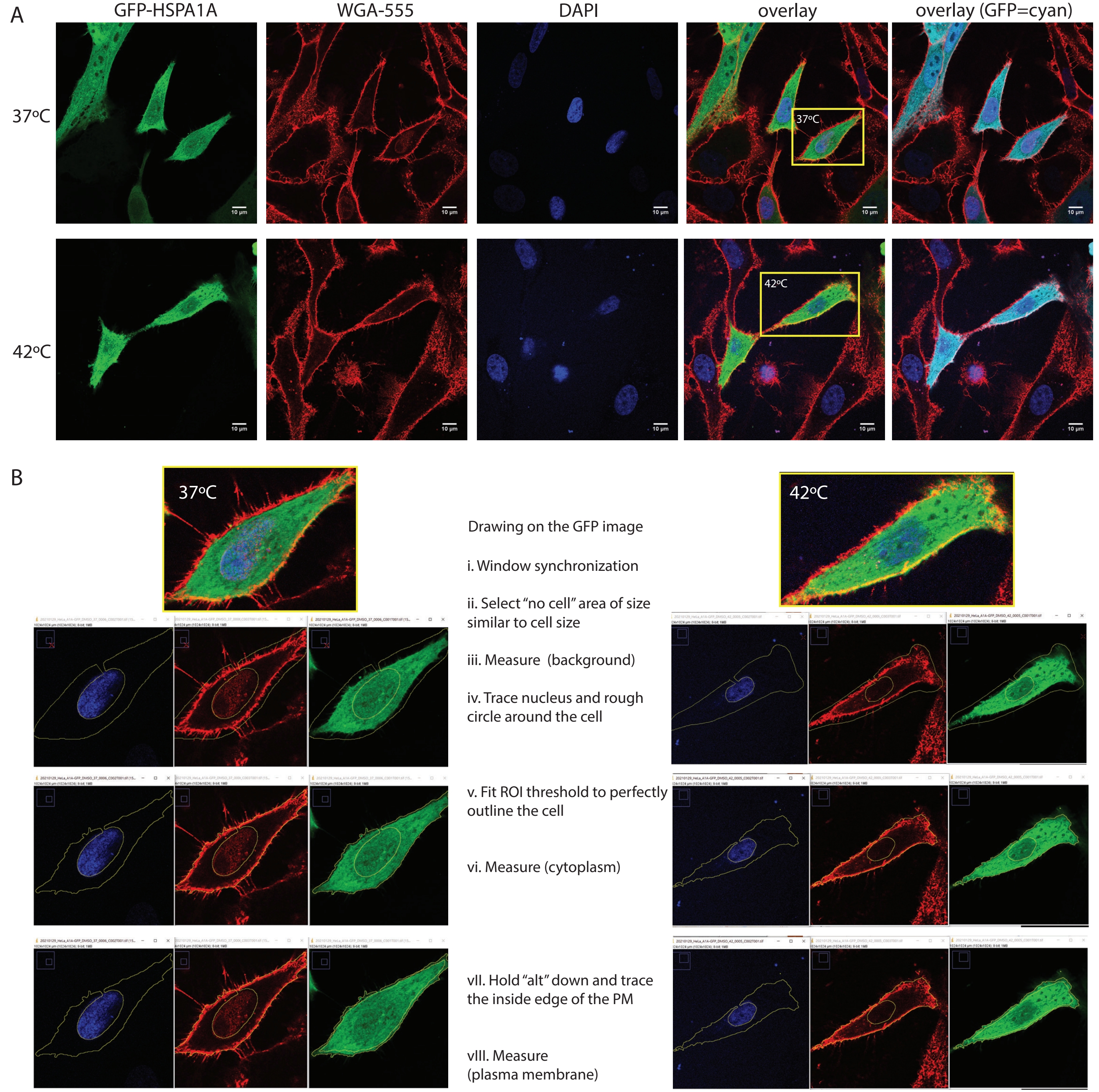

Calculate corrected total cell fluorescence (CTCF) = Integrated Density – (Area of Region of Interest * Fluorescence of background reading)

Figure S1

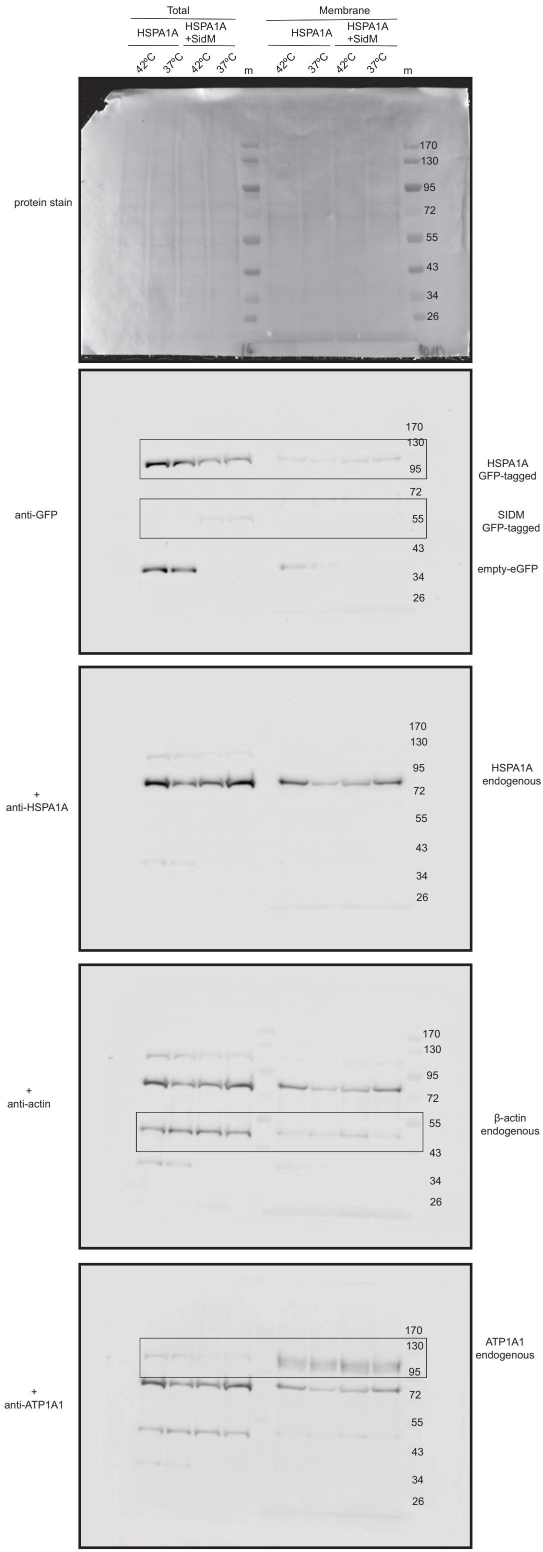
Total
Membrane
HSPA1A
HSPA1A +SidM
HSPA1A
HSPA1A +SidM
42ºC 37ºC 42ºC 37ºC m 42ºC 37ºC 42ºC 37ºC m
protein stain
170 130 95 72 55 43 34 26
anti-GFP
HSPA1A GFP-tagged
SIDM GFP-tagged
empty-eGFP
170 130 95 72 55 43 34 26
+ anti-HSPA1A
HSPA1A endogenous
170 130 95 72 55 43 34 26
+ anti-actin
β-actin endogenous
170 130 95 72 55 43 34 26
+ anti-ATP1A1
ATP1A1 endogenous
170 130 95 72 55 43 34 26

Figure S2A

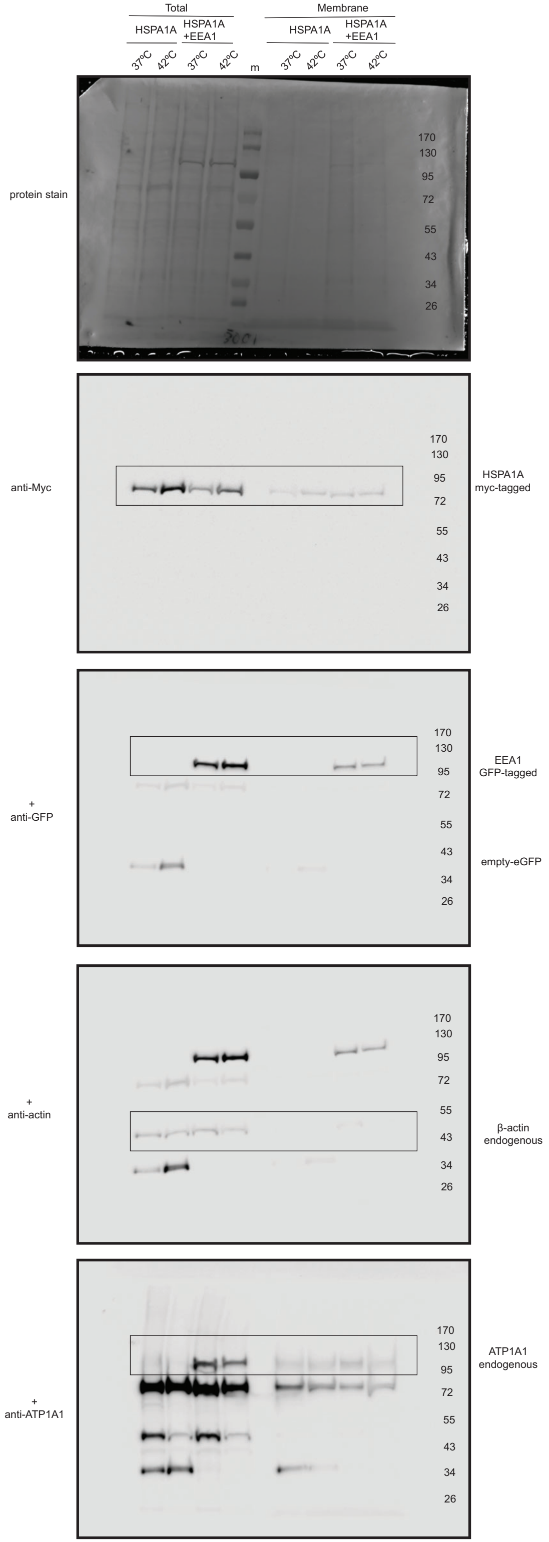
Total
Membrane
HSPA1A
HSPA1A +EEA1
HSPA1A
HSPA1A +EEA1
37°C 42°C 37°C 42°C m 37°C 42°C 37°C 42°C
protein stain
170 130 95 72 55 43 34 26
anti-Myc
HSPA1A myc-tagged
170 130 95 72 55 43 34 26
+ anti-GFP
EEA1 GFP-tagged
empty-eGFP
170 130 95 72 55 43 34 26
+ anti-actin
β-actin endogenous
170 130 95 72 55 43 34 26
+ anti-ATP1A1
ATP1A1 endogenous
170 130 95 72 55 43 34 26

Figure S2B

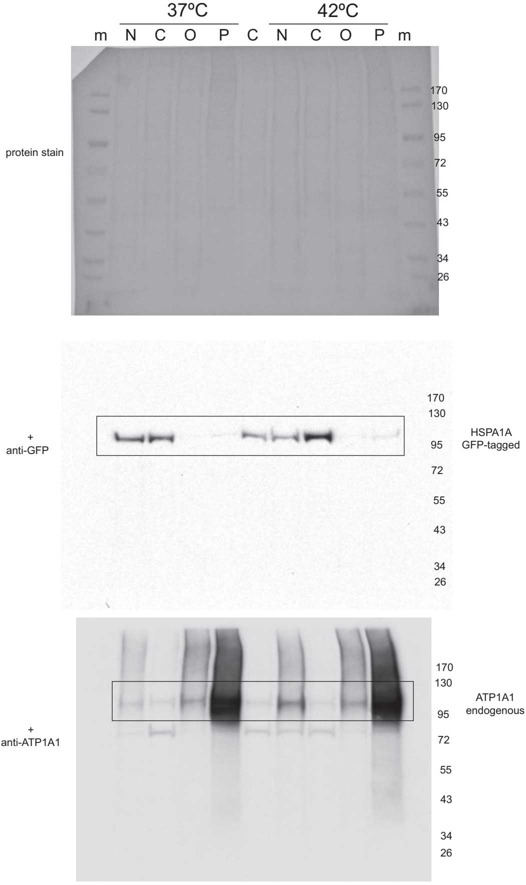

N= Nucleus; C= Cytosol; O= Organelle Membranes; P= Plasma Membrane

Figure S3A

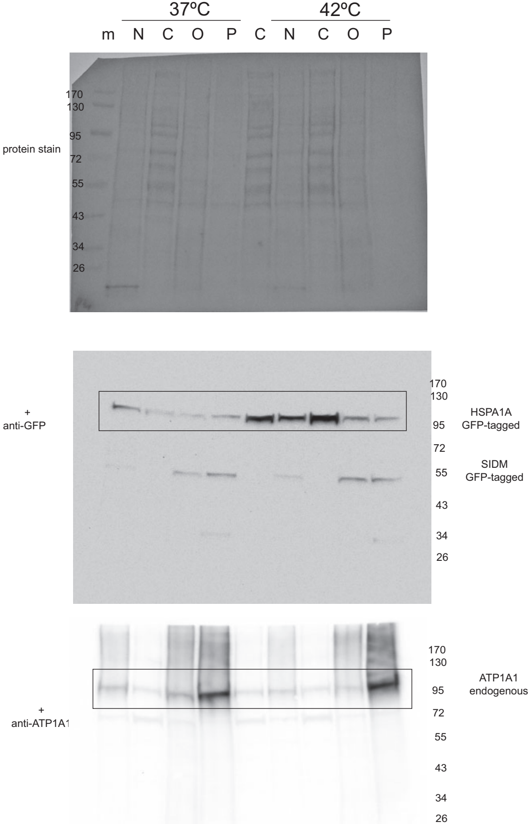

N= Nucleus; C= Cytosol; O= Organelle Membranes; P= Plasma Membrane

Figure S3B

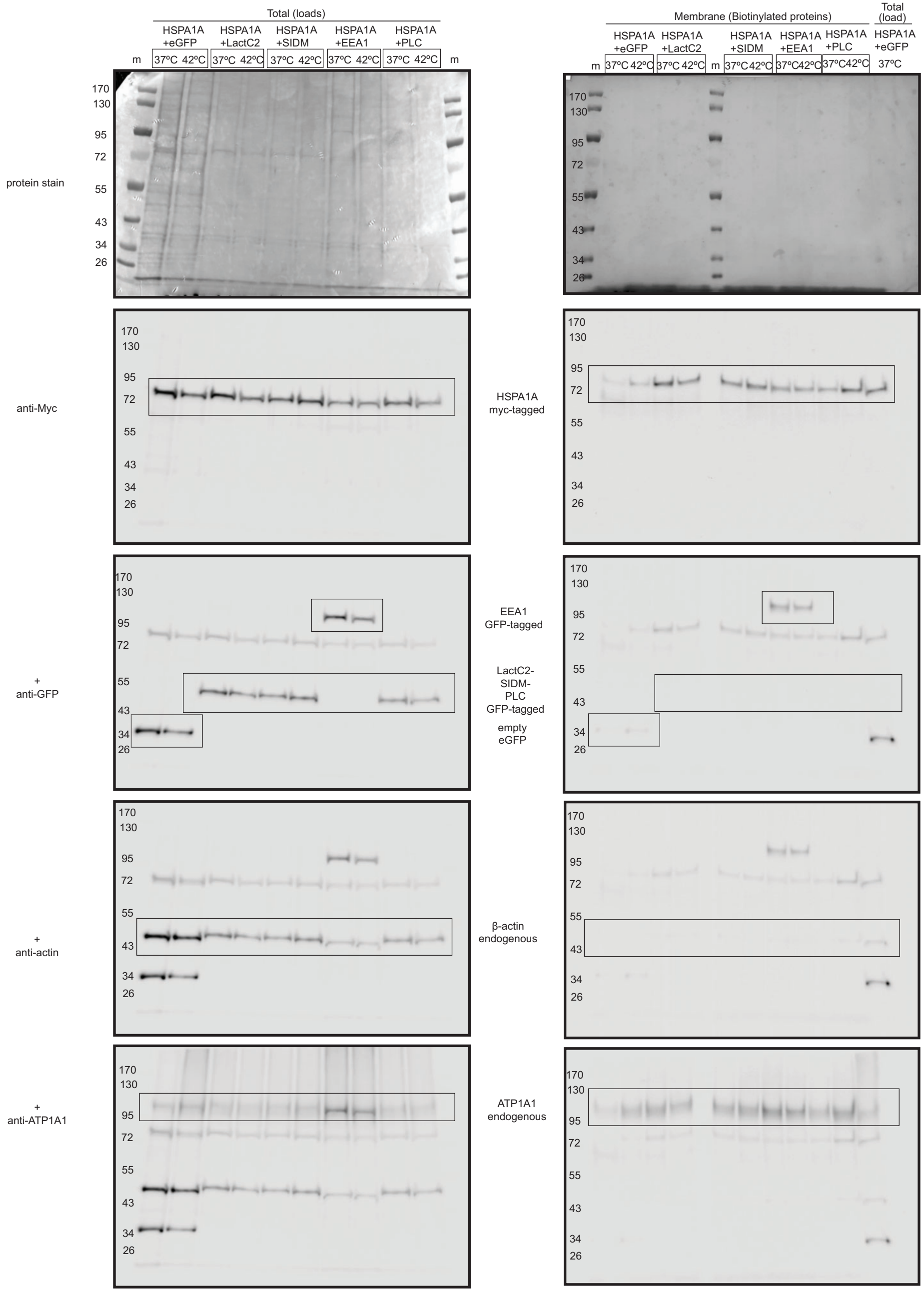
Total (loads)
HSPA1A +eGFP
HSPA1A +LactC2
HSPA1A +SIDM
HSPA1A +EEA1
HSPA1A +PLC
37ºC 42ºC
m
Membrane (Biotinylated proteins)
Total (load)
HSPA1A +eGFP
37ºC
protein stain
anti-Myc
+ anti-GFP
+ anti-actin
+ anti-ATP1A1
HSPA1A myc-tagged
EEA1 GFP-tagged
LactC2- SIDM- PLC GFP-tagged
empty eGFP
β-actin endogenous
ATP1A1 endogenous
170
130
95
72
55
43
34
26

Figure S4

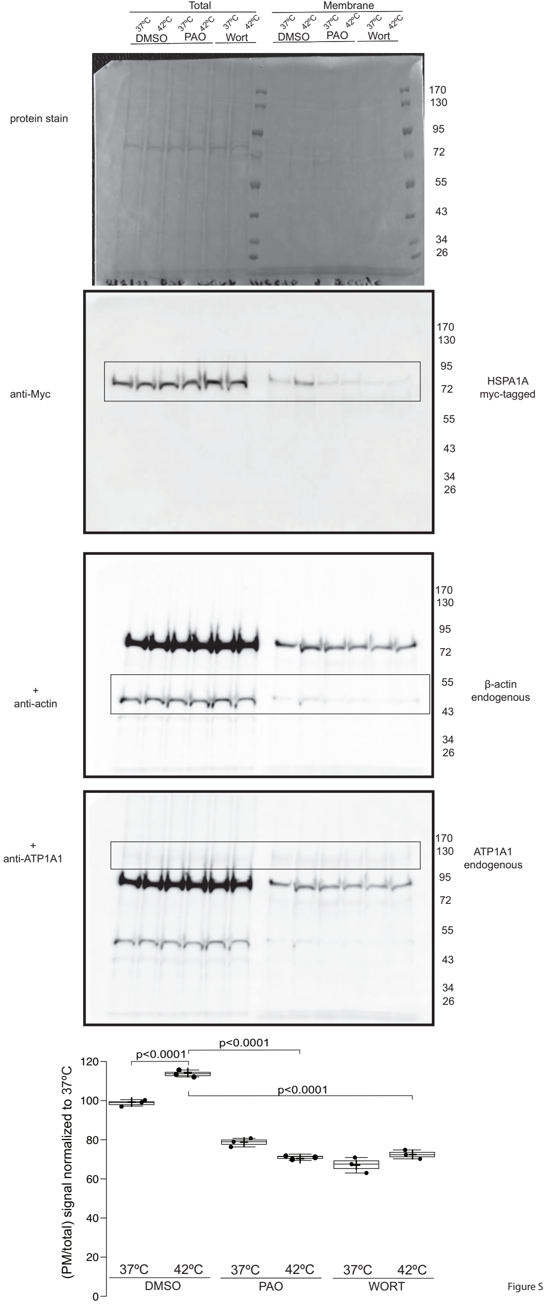
Total
Membrane
37ºC 42ºC 37ºC 42ºC 37ºC 42ºC
DMSO PAO Wort
37ºC 42ºC 37ºC 42ºC 37ºC 42ºC
DMSO PAO Wort
protein stain
170
130
95
72
55
43
34
26
anti-Myc
HSPA1A
myc-tagged
+
anti-actin
β-actin
endogenous
+
anti-ATP1A1
ATP1A1
endogenous
p<0.0001
p<0.0001
p<0.0001
(PM/total) signal normalized to 37ºC
0
20
40
60
80
100
120
37ºC
42ºC
DMSO
PAO
WORT

Figure S5

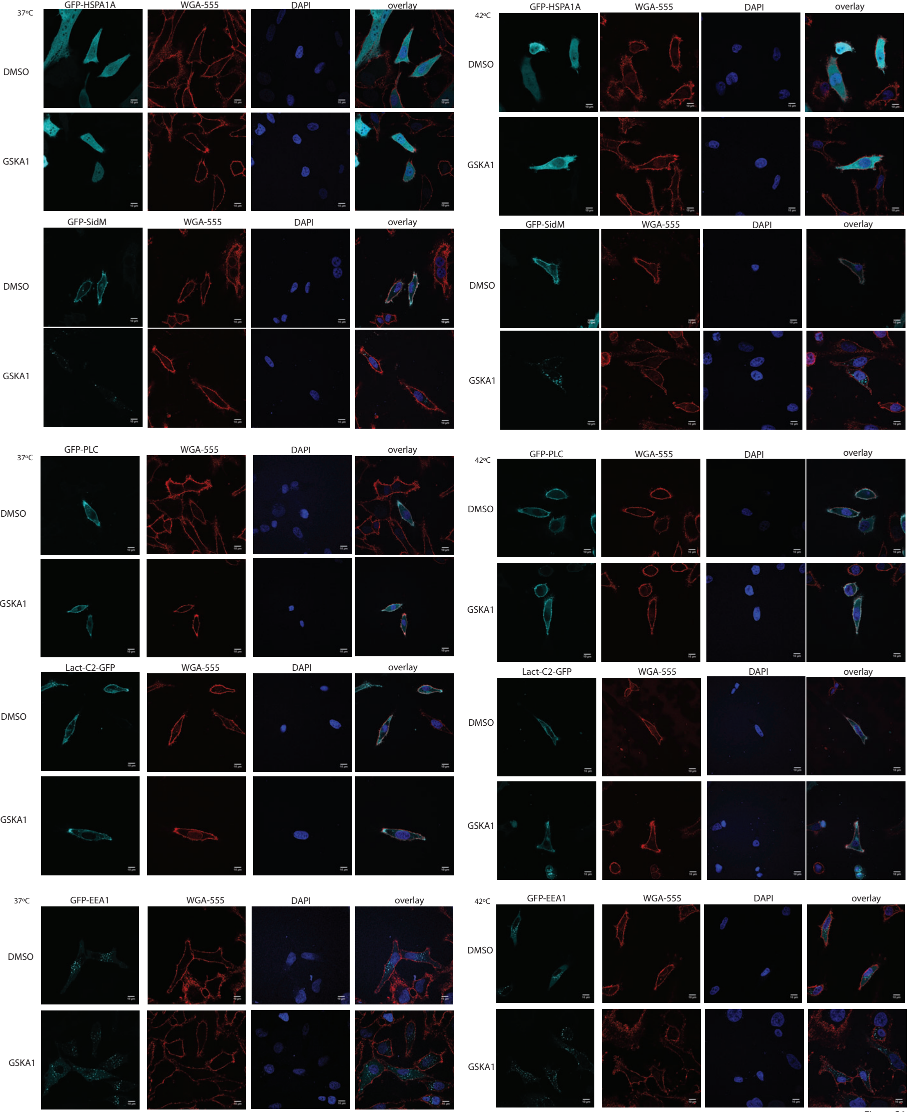
37ºC
GFP-HSPA1A
WGA-555
DAPI
overlay
DMSO
GSKA1
GFP-SidM
WGA-555
DAPI
overlay
DMSO
GSKA1
42ºC
GFP-HSPA1A
WGA-555
DAPI
overlay
DMSO
GSKA1
GFP-SidM
WGA-555
DAPI
overlay
DMSO
GSKA1
37ºC
GFP-PLC
WGA-555
DAPI
overlay
DMSO
GSKA1
Lact-C2-GFP
WGA-555
DAPI
overlay
DMSO
GSKA1
42ºC
GFP-PLC
WGA-555
DAPI
overlay
DMSO
GSKA1
Lact-C2-GFP
WGA-555
DAPI
overlay
DMSO
GSKA1
37ºC
GFP-EEA1
WGA-555
DAPI
overlay
DMSO
GSKA1
42ºC
GFP-EEA1
WGA-555
DAPI
overlay
DMSO
GSKA1

Figure S6

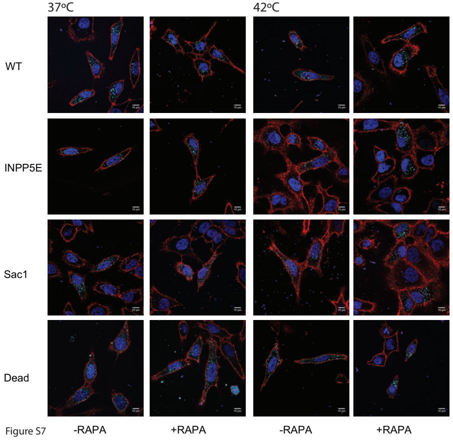

Figure S7
